# Effect of pre-stroke use of ACE inhibitors on ischemic stroke severity

**DOI:** 10.1186/1471-2377-5-10

**Published:** 2005-06-10

**Authors:** Magdy Selim, Sean Savitz, Italo Linfante, Louis Caplan, Gottfried Schlaug

**Affiliations:** 1 Department of Neurology, Beth Israel DeaconessMedical Center, Boston, USA; 2 Interventional Neuroradiology, Jackson Memorial Hospital, Miami, USA

## Abstract

**Background:**

Recent trials suggest that angiotensin-converting enzyme inhibitors (ACEI) are effective in prevention of ischemic stroke, as measured by reduced stroke incidence. We aimed to compare stroke severity between stroke patients who were taking ACEI before their stroke onset and those who were not, to examine the effects of pretreatment with ACEI on ischemic stroke severity.

**Methods:**

We retrospectively studied 126 consecutive patients presenting within 24 hours of ischemic stroke onset, as confirmed by diffusion-weighted magnetic resonance imaging (DWI). We calculated the NIHSS score at presentation, as the primary measure of clinical stroke severity, and categorized stroke severity as mild (NIHSS [less than or equal to] 7), moderate (NIHSS 8–13) or severe (NIHSS [greater than or equal to] 14). We analyzed demographic data, risk-factor profile, blood pressure (BP) and medications on admissions, and determined stroke mechanism according to TOAST criteria. We also measured the volumes of admission diffusion- and perfusion-weighted (DWI /PWI) magnetic resonance imaging lesions, as a secondary measure of ischemic tissue volume. We compared these variables among patients on ACEI and those who were not.

**Results:**

Thirty- three patients (26%) were on ACE-inhibitors. The overall median baseline NIHSS score was 5.5 (range 2–21) among ACEI-treated patients vs. 9 (range 1–36) in non-ACEI patients (p = 0.036). Patients on ACEI prior to their stroke had more mild and less severe strokes, and smaller DWI and PWI lesion volumes compared to non-ACEI treated patients. However, none of these differences were significant. Predictably, a higher percentage of patients on ACEI had a history of heart failure (p = 0.03). Age, time-to-imaging or neurological evaluation, risk-factor profile, concomitant therapy with lipid lowering, other antihypertensives or antithrombotic agents, or admission BP were comparable between the two groups.

**Conclusion:**

Our results suggest that ACE-inhibitors may reduce the clinical severity of stroke, as measured by NIHSS score. Further, larger-scale, prospective studies areneeded to validate our findings, and to elucidate the mechanism(s) of ACEImediated benefits in patients with ischemic stroke.

## Background

Data from the heart outcomes prevention evaluation study (HOPE) suggest that angiotensin-converting enzyme inhibitors (ACEI) are effective in prevention of ischemic stroke, as measured by reduced stroke incidence in subjects randomized to treatment with ACEI [1]. In this trial, the use of the ACEI, ramipril, resulted in a 32% reduction in ischemic stroke risk despite minimal reduction in blood pressure (BP) [1], leading some to suggest that ACEI may also exert direct neuroprotective effects.

To further elucidate if ACEI have potential neuroprotective effects, we tested whether their use prior to ischemic stroke onset might also reduce the severity of stroke. We examined clinical and admission magnetic resonance imaging (MRI) data from patients with ischemic stroke to determine the effects of prestroke use of ACEI on stroke severity.

## Methods

### Study design and patient selection

We retrospectively reviewed our prospectively collected stroke database over a 30-month period from 1998 to 2000, and identified consecutive patients who presented with acute ischemic stroke within 24 hours of onset and had DWI/PWI upon presentation. Onset time was defined, as the last time the patient was known to be in his/her usual state of health. The diagnosis of ischemic stroke was confirmed by diffusion-weighted imaging (DWI) showing evidence of acute cerebral infarction, combined with serial neurological examinations performed by stroke-trained neurologists. We included patients who had received thrombolytic, endovascular or experimental neuroprotective treatment. We only excluded patients who had transient ischemic attacks (TIAs), in whom DWI/PWI was negative.

### Data collection and assessments

We retrieved the following data for each patient: (1) demographics; (2) risk factors for stroke, i.e. hypertension (HTN), diabetes mellitus (DM), hyperlipidemia, coronary artery disease (CAD), atrial fibrillation (AF), heart failure (CHF), history of TIA and smoking, as reported by the patient andhis/her family; (3) vital signs at presentation (BP and temperature); (4) blood glucose level at admission; (5) medications upon admission, with particular attention to antiplatelets, anticoagulants, lipid-lowering agents, and antihypertensives including ACEI. We did not collect information about the duration of medication(s) use, daily use or compliance. Patients and families were only questioned about patient's use of medication(s), including ACEI, in the week before stroke; (6) the baseline National Institute of Health Stroke Scale (NIHSS) score [2], which was recorded by stroke-trained neurolgistscertified in the application of NIHSS at admission; and (7) time from strokedetection to imaging.

### Outcome measures

We used the NIHSS score at presentation as the primary measure of clinical stroke severity, and categorized stroke severity as mild (NIHSS score = 7), moderate (NIHSS score 8–13) or severe (NIHSS score = 14). We measured the total DWI and PWI lesion volumes, as secondary radiological measures of stroke severity, in 110/126 patients. All MRI studies were performed on a Siemens Medical Systems Vision 1.5-T MR whole body scanner with echoplanar imaging capabilities. An experienced researcher blinded to clinical data and patient's identity, performed MRI measurements. The volume of the perfusion abnormality was measured on relative Mean Transit Time (rMTT) maps. The specific MRI sequence parameters, imaging processing and volumetric analysis are described in details in previous publications [3,4]. We classified stroke mechanisms, after completing the diagnostic work-up, according to the Trial of Org 10172 in Acute Treatment (TOAST) criteria [5].

### Statistical analysis

We divided patients into 2 groups, those who were taking ACEI before their stroke onset and those who were not. We compared inter-group differences between individual categorical variables by using student's *t*-test or Wilcoxon rank sum test for continuous variables, and Fisher's exact test forcategorical comparisons, as appropriate. We compared the median baseline NIHSS scores with the Mann-Whitney U test to evaluate the severity of the stroke in both groups. The Cochran-Mantel-Haenszel row mean score rank test, adjusted for various confounding variables (age, sex, risk factors, use of concomitant medications, time-from-stroke-to-evaluation, and stroke mechanism/subtype) was used to control for the differences in NIHSS scores between ACEI and non-ACEI users. A *p*-value of < 0.05 was considered statistically significant for all analyses.

## Results

### Patient characteristics (demographic and clinical Features)

A total of 126 patients met all of our inclusion and none of the exclusion criteria, and were included in subsequent analyses. Approximately, 26% (33 patients) were on ACEI before stroke onset. Fourteen were taking lisinopril (20 to 40 mg per day), 13 enalapril (10 to 40 mg per day), 5 captopril (75 to 150 mg per day) and 1 accupril (40 mg per day). None of our patients was taking perindopril or ramipril, or a combination of different ACEI. Table 1 summarizes the demographic and clinical features of patients in both ACEI- and non ACEI-treated groups. There were no significant differences in the mean age or sex distribution between the 2 groups. Slightly higher percentages of ACEI-treated patients had history of HTN, DM, hyperlipidemia, and smoking in comparison to the non-ACEI group. A slightly higher percentage of non-ACEI group reported history of prior TIA. However, none of these differences were statistically significant. There was a trend towards a higher frequency of cardiac disease among ACEI-treated patients. This was mostly driven by a significantly higher percentage of heart failure among ACEI-treated patients.

**Table 1:** Comparison of demographic and clinical features between ACEI- and non ACEI-treated groups.

Number of patients	ACEI group 33 (26%)	Non-ACEI group 93 (74%)	p-value
Sex (women/men)	13/20	43/50	
• % women	39%	46%	0.55
• % men	61%	54%	0.55
Mean age (year) ± SD	73 ± 11	70 ± 13	0.23
Risk factors:			
History of hypertension	78%	65%	0.19
History of diabetes	32%	24%	0.36
History of hyperlipidemia	28%	22%	0.48
History of cardiac disease	39%	21%	0.06
• CHF	23%	9%	0.03*
• AF	10%	7%	0.69
• CAD	6%	5%	0.98
History of smoking	16%	11%	0.53
History of prior TIA	10%	16%	0.39
Concomitant medications:			
Antiplatelets	37%	41%	0.68
Anticoagulants	12%	10%	0.74
Statins	20%	21%	0.99
Other BP lowering agents	56%	52%	0.84
• Diuretics	25%	18%	0.45
• B-blockers	18%	27%	0.35
• Ca^++ ^blockers	13%	13%	0.99
• ARB	0%	4%	0.57
Time from stroke-to evaluation			
• 0–6 h	61%	63%	0.83
• 6–25 h	39%	37%	0.83
Clinical features:			
NIHSS score, median	5.5	9	0.036*
SBP (mean ± SD), mmHg	162 ± 27	158 ± 31	0.35
DBP (mean ± SD), mmHg	84 ± 16	81 ± 20	0.38
Temperature (mean ± SD), F°	97 ± 7	98 ± 6	0.34

A roughly equal percentage of patients in both groups presented to our emergency room and were imaged within 6 hours from stroke onset (61% in ACEI-treated patients vs. 63% in non-ACEI group; p = 0.83). The mean time from stroke-symptom onset to evaluation was 10.9+5.2 h in ACEI-treated patients vs. 11.3+6.4 h in non-ACEI group (p = 0.62). There were no significant differences in admission temperature (97+7 F° vs. 98+6 F°; p = 0.34) or glucose levels (137+23 mg/dL vs. 144+29 mg/dL; p = 0.26) between the 2 groups. The mean SBP upon admission was 162+27 mmHg in ACEI-treated patients vs. 158+31 mmHg in non-ACEI group, and the mean DBP was 84+16 vs. 81+20 mmHg. None of these differences were statistically significant.

A roughly equal percentage of patients in each group were using antiplatelets, anticoagulants, statins and other BP lowering agents. Similarly, the frequency of using other classes of antihypertensive agents was not significantly different in either group. Four patients were taking angiotensin receptor blockers (ARBs) at the time of their stroke. None of these 4 patients was on ACEI. They were all included in non ACEI-treated group for purposes of statistical analysis.

### Patient outcomes

Approximately, 48% of ACEI-treated patients had baseline NIHSS score = 7 compared with 40% of non-ACEI group (p = 0.42); 28% had NIHSS score between 8 – 13 vs. 20% in non-ACEI group (p = 0.46); and 24% had NIHSS score = 14 vs. 40% in non-ACEI users (p = 0.18).

**Figure 1:**
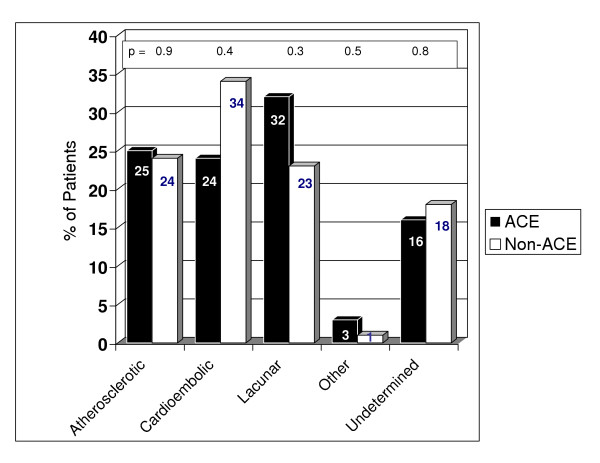
Comparison of stroke mechanisms between ACEI- and non ACEI-treated patients.

Figure 1 shows the distribution of stroke mechanisms, according to TOAST criteria, among ACEI- and non ACEI-treated patients. The stroke mechanisms were roughly equivalent in both groups. Although, cardioembolic cause was more frequent among non ACEI-treated patients (34% vs. 24%) and lacunar etiology was more commonly seen among patients who were taking ACEI prior to stroke onset (32% vs. 23%), these differences were not statistically significant.

The overall median NIHSS score at admission was significantly lower in ACEI-treated patients (5.5; range 2 – 21) than in non-ACEI patients (9; range 1 – 36; p = 0.036). This difference remained statistically significant after controlling for confounding variables, such as history of hypertension, TIA, DM hyperlipidemia and cardiac disease, including CHF, stroke mechanism, onsetto- evaluation time, and concomitant medications, using the Cochran-Mantel-Haenszel row mean score test using ranks adjusted for these covariates (p = 0.042). Similarly, the median NIHSS score at admission was lower in the ACEI group when the analysis was limited to patients with non-lacunar strokes (8.5 vs. 12; p = 0.03) or to those who presented within 6 hours of stroke symptom onset (6 vs. 8; p = 0.046).

The DWI/PWI lesions volumetric measurements were performed in 87% of the patients (110/126). Sixteen patients were not included in MRI data analyses because their images were of poor quality to allow adequate quantitative measurements. As table 2 indicates, there were no significant differences between both groups with regard to the mean diffusion, perfusion or perfusion-diffusion (mismatch) lesion volumes.

**Table 2:** Comparison of MRI between ACEI- and non ACEI-treated group

	ACEI group	Non-ACEI group	p-value
DWI lesion volume (mean ± SD), cm^3^	25.2 ± 23.4	28.7 ± 25.0	0.55
PWI lesion volume (mean ± SD), cm^3^	72.6 ± 56.6	75.1 ± 68.5	0.86
Mismatch (PWI – DWI) volume	47.6 ± 39.5	46.6 ± 28.2	0.93

## Discussion

We found that the baseline NIHSS score was lower in patients who were taking ACEI prior to their stroke compared to those who were not taking ACEI at the time of stroke onset. The NIHSS is accepted widely for measuring acute stroke deficits to assess the degree of severity of neurological deficits from stroke and its reliability has been tested in several clinical trials [6-8].

We found no difference in admission BP between ACEI and non-ACEI users, suggesting that the beneficial effects of ACEI use may not be directly related to their BP-lowering effect. This is concordant with the results from the HOPE trial [1].

Several factors could potentially account for the observed difference in baseline NIHSS between ACEI and non-ACEI groups, such as differences in risk factors, stroke mechanism/subtype, or baseline hemodynamic parameters. The risk factors that influence ACEI use, such as history of HTN, CHF and DM, were dissimilarly distributed between the two groups, and their impact on stroke type and severity cannot be entirely excluded. However, ourfindings are unlikely to be related to differences in baseline risk factor profile between the ACEI- and non-ACEI treated patients since patients who were on ACEI had a higher prevalence of HTN, DM and heart failure, which may have biased our data toward higher stroke severity in ACEI-treated patients, and thus limited our ability to detect larger differences in favor of ACEI use. It is possible that ACEI use reflected a greater degree of medical attention and more aggressive risk factor reduction in these patients, which subsequently lessened stroke severity.

Recent studies have shown that TIAs before stroke can induce tolerance (ischemic preconditioning) to subsequent strokes by raising the threshold of brain tissue vulnerability, which results in smaller infarct volumes, and better recovery [9-11]. We found no significant differences in the frequency of prior history of TIAs, as reported by the patient or his/her family, between the ACEI and non-ACEI treated patients. In fact, a slightly higher percentage of patients in the non-ACEI group reported history of TIAs prior to their presenting stroke.

Some epidemiological studies show that greater stroke severity at onset is associated with a shorter interval between symptom onset and time to emergency department arrival [12], suggesting that the observed difference in baseline NIHSS could be attributed to dissimilar distribution of patients' arrival time to the hospital. However, a roughly identical proportion of patients in both groups presented to our hospital within 6 hours of stroke-symptom onset.

Since the observed beneficial effect of ACEI on stroke severity could potentially be secondary to ACEI effects on stroke mechanism, we examined the impact of ACEI use on stroke mechanism using TOAST criteria. We found a greater preponderance of lacunar strokes among ACEI-treated patients and cardioembolic strokes among non-ACEI patients. However, these differences were not significant and the difference in baseline NIHSSS remained significant even after excluding patients with non-lacunar strokes from analysis. This suggests that ACEI use did not seem to influence stroke mechanism in our cohort of patients, and that our findings were unrelated to the higher frequency of lacunar strokes among ACEI-treated patients. Similarly, the beneficial effect of ACEI in our patients is unlikely to be related to other concomitant treatments. Although, several patients in both groups were on statins, antithrombotics and other antihypertensive agents, we found no significant difference between ACEI- and non ACEI-treated patients receiving any of these classes of drugs. Finally, it is noteworthy that the difference in baseline stroke severity between ACEI and non-ACEI groups remained statistically significant after adjusting for the above confounding variables.

A recent prospective observational study of 507 patients with first-ever ischemic stroke showed that treatment with ACEI at the time of stroke onset is associated with reduced plasma concentration of C-reactive protein and better long-term outcomes [13], suggesting that ACEI may have anti-inflammatory properties and reduce the acute-phase inflammatory response after stroke onset. There are several other potential mechanisms by which ACEI may provide benefit to stroke patients. Experimental data suggest that the rennin angiotensin system modulates the atherosclerotic process, and that angiotensin II exerts pro-inflammatory actions in the vascular wall, which induce the production of reactive oxygen species and hydroxyl radicals, cytokines and adhesion molecules [14-19]. Angiotensin converting enzyme inhibitors could provide neuroprotection via blockade of angiotensin II-mediated endothelial dysfunction, lipid peroxidation and subsequent oxidative stress, and vascular smooth muscle intracellular calcium accumulation and hypertrophy [14-20]. Furthermore, ACEI may help maintain homeostatic balance of fibrinolytic and procoagulant factors [21] and increase cerebral blood flow [22]. Recent studies using transcranial Doppler ultrasonography have shown that perindopril can improve the cerebral vasomotor reactivity in patients with lacunar infarcts beyond any effect on BP [22], and that treatment with quinapril can ameliorate cerebrovascular reactivity caused by methionine-induced hyperhomocysteinemia in healthy volunteers [23].

We found that ACE I use had no effect on MRI measures of ischemic lesion volume. This discrepancy between the beneficial effects of ACEI on clinical, but not radiological, measures of stroke severity is reconcilable since the correlation between infarct volume and NIHSS is only moderate, particularly in non-dominant hemispheric strokes [24-26]. We explored the possibility that the lower NIHSS scores in ACEI-treated patients might be secondary to a higher percentage of non-dominant hemispheric strokes in this group [26]. However, we found no significant difference in the preponderance of non-dominant hemispheric strokes between the 2 groups (data not reported). Infarct location, not only size, is also an important determinant of the severity of clinical deficits and our small sample size may have limited our ability to detect a difference in favor of ACEI.

We acknowledge that our study has inherent limitations imposed by its retrospective nature, non-randomization of treatment allocation and small sample size. The small number of ACEI-treated patients does not allow us to test for possible differences among the various ACEIs or dose regimens. Similarly, we cannot be certain of the duration of treatment or compliance with daily use of ACEI in our patients. We used an arbitrary cut-off for NIHSS scores to categorize stroke severity. It is possible that different cut-off values could lead to different results. Most importantly, our study lacks follow-up data regarding the effect of ACEI use on long-term outcomes since a large percentage of our patients were either enrolled in experimental neuroprotective trials or treated with thrombolysis upon presentation.

## Conclusion

Our results show that pre-stroke use of ACEI is associated with milder stroke severity, as assessed by NIHSS score. Our findings need to be prospectively validated in larger-scale randomised studies, and the mechanism(s) of ACEI-mediated benefits in patients with ischemic stroke need to be elucidated.

## Competing interests

The author(s) declare that they have no competing interests.

## Authors' contributions

MS designed the study, collected and analyzed data, wrote the paper, and carried out critical revision of the manuscript. SS collected and analyzed data, and reviewed the manuscript. IL collected data and reviewed the manuscript. LC assisted with data interpretation and critically reviewed the manuscript. GS collected data, and carried out critical revision of the manuscript.

## Pre-publication history

The pre-publication history for this paper can be accessed here:


